# A Patient with Splenic Artery Aneurysm Rupture and the Importance of Rapid Sonography in the ED

**DOI:** 10.1155/2010/893606

**Published:** 2010-06-29

**Authors:** Masayuki Iyanaga, Susan Watts, Takeshi Kasai

**Affiliations:** ^1^Department of Emergency Medicine, Texas Tech University Health Sciences Center, El Paso TX 79905, USA; ^2^Department of Emergency Medicine, Kameda Medical Center, Chiba Prefecture, 296-8602, Japan

## Abstract

We report a case of a splenic artery aneurysm rupture presenting with shock which required timely embolization therapy. This case demonstrates how the rapid use of bedside ultrasound by emergency department (ED) physicians can help identify the cause of shock and, therefore, initiate appropriate treatment quickly even if the cause is rare, as in this case.

## 1. Introduction

Emergency physicians often care for patients who are clinically in shock and it can be difficult to determine the cause of the symptoms. In some cases, bedside ultrasound performed by the physician can provide clues that will quickly lead to a definitive diagnosis and appropriate management which can save a life.

## 2. Case Report

A 76-year-old Asian male was brought to the emergency department (ED) from a nearby hospital clinic. The man had had an episode of syncope and was found kneeling in a hallway near the hospital clinic where he had a follow-up appointment for a thyroid mass. Nurses and a doctor from the clinic responded to the scene, and they said the man reported left flank pain, dizziness, and one episode of vomiting just after falling. His vital signs measured by nurses at the scene included BP 80/40, PR 40, and SpO2 92% (on room air). They reported that his physical examination at that time showed anemic conjunctivae in both eyes but equal and reactive pupils, his chest was clear to auscultation bilaterally, and that heart sounds were unremarkable. Otherwise his physical exam was considered noncontributory. They gave him oxygen by nasal cannula (3 L/min) and 750 mL of normal saline intravenously (IV), but his hypotension did not improve. 

 Consequently, the man was sent emergently to the ED about 30 minutes after the incident. His vital signs on arrival to the ED were BP 82/not palpable, PR 40, RR 18, T 36.3, and SpO2 84% (oxygen 10 L/min). His past medical history was not significant except for hypertension and a left thyroid mass. His past surgical history included left inguinal hernia repair. He took two antihypertensive medications, doxazosin 1mg QD and nifedipin 40 mg QD. He denied allergies to medications or foods. 

 Significant laboratory studies included hemoglobin 11.9 gm/dL, hematocrit 37.4%, WBC 5,700/*μ*L, and platelets 115,000/*μ*L. Electrolytes, liver and renal function tests, and cardiac enzymes were all normal. Arterial blood gas (ABG) results were PaO_2_ 157.1 mm Hg, PaCO_2_ 42.6 mm Hg, and HCO_3_ 25.3 mEq/L under oxygen 3 L/min. His CXR was within normal limits while his EKG showed sinus bradycardia without signs of cardiac ischemia. 

 On physical exam, mild tenderness in the left upper quadrant was detected. With additional questioning, the man related that before he fell near the clinic, he had tripped on the stairs, fell down the stairs about 3 feet, then hit his left torso against the floor. Sonography was performed at the bedside by a physician, and there was no evidence of an abdominal aortic aneurysm or pericardial effusion. However, there was mild fluid retention at Morison's pouch and in the vesicorectal pouch. At this point, we had ruled out AAA and cardiac tamponade and the new preliminary diagnosis became abdominal trauma and hemiperitoneum of an unknown cause. 

**Figure 1:**
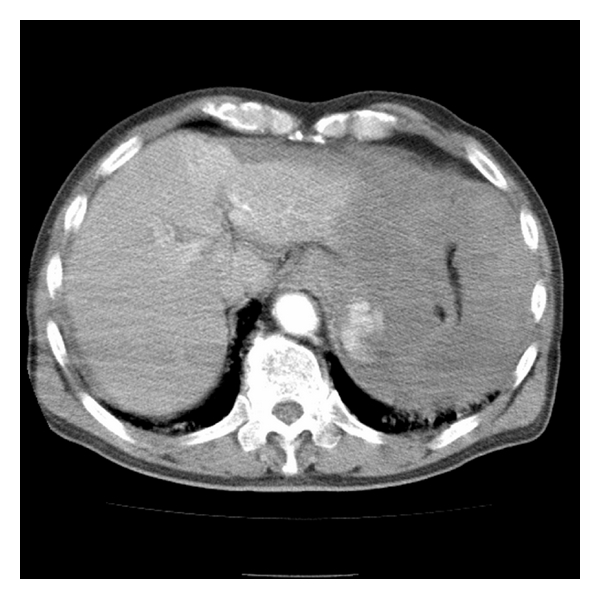
Transverse plane of helical CT of abdomen with IV contrast shows extravasation at splenic hilum and fluid around spleen.

**Figure 2:**
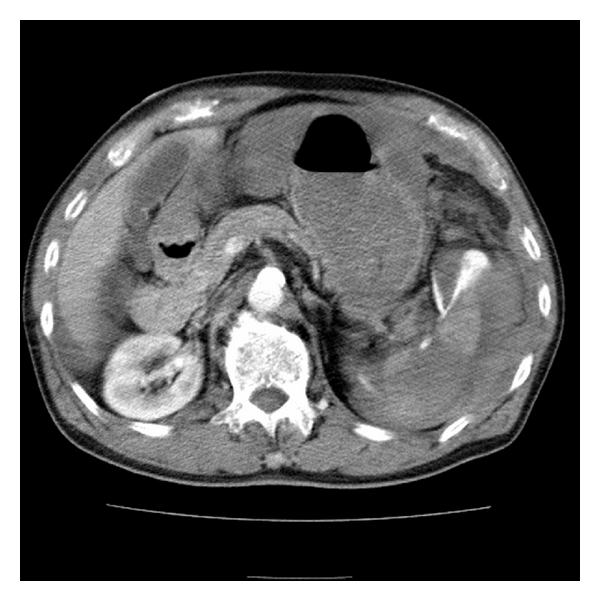
Transverse plane of helical CT of abdomen with IV contrast shows extravasation at splenic hilum and fluid around spleen.

**Figure 3:**
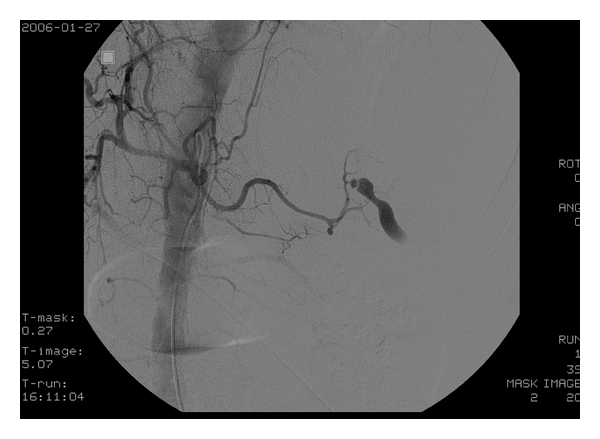
Arteriogram of celiac artery displays a small aneurysm rupture at a branch of splenic artery.

**Figure 4:**
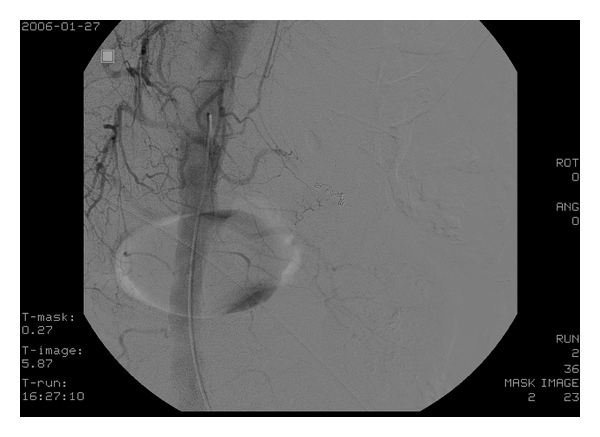
Arteriogram after embolization to a splenic aneurysm demonstrates no extravasation.

 After starting a blood transfusion, the patient was taken for chest and abdominal CT scans which showed severe extravasation at the hilum of the spleen along with poor contrast effect, implying splenic artery injury (Figures 1 and 2). Surgery was consulted and they took the patient to angiography where the celiac artery was visualized with contrast. A splenic artery aneurysm with extravasation was identified (Figure 3) and subsequently embolized with a coil and gel-form substance (Figure 4). The patient was stabilized and admitted to the ICU.

## 3. Discussion

Splenic artery aneurysm is the most common form of the splanchnic artery aneurysm, and its prevalence rate is 0.04% to 0.1% at autopsy [1]. Most of the aneurysms are caused by degenerative atherosclerosis or portal hypertension. The risk of rupture is about 5% [2], but in a study at a Taiwanese ED, 4 of 7 patients with splenic artery aneurysm developed an aneurysm rupture [3]. Patients with a ruptured aneurysm can present with acute abdomen, hypotension, and hemorrhagic shock. The other symptoms include upper epigastric pain or left upper quadrant pain with radiation to left shoulder [2]. The risk of rupture is higher in pregnant women and if the aneurysm is more than 2 cm in diameter [2]. The mortality rate in patients with ruptured aneurysm was 20% in the study at Mayo Clinic [2], while the rate was 25% in the Taiwanese study though the number of patients was small [3]. The double rupture phenomenon may occur, in which the aneurysm first ruptures into the lesser sac with mild clinical symptoms then the blood overflows into peritoneal cavity through the Winslow foramen with hemorrhagic shock [2]. Splenic artery aneurysm is usually noted in CT or angiography as an incidental finding. High-resolution CT or angiography is required for the definitive diagnosis [2]. Ruptured splenic artery aneurysm is treated with splenectomy or percutaneous embolization [2]. 

 This case was challenging because the differential for syncope in the elderly is large, and the incomplete details of the history of the patient's illness provided little direction. The vital signs at presentation could be attributed to hemorrhagic shock but could also be explained by an overdose of antihypertensives. Bedside ultrasound pointed the way to the definitive diagnosis by showing the fluid accumulation in the peritoneal cavity, thereby reducing the possibilities. Without bedside ultrasound, it would have taken more time for the patient to get the appropriate treatment. In addition, this case demonstrates that for patients with an unknown cause of shock, it is very important for emergency physicians to have hemorrhagic shock, including the rare splenic artery aneurysm, in their differential diagnosis list. 

 This case illustrates the need for a goal-directed ultrasound policy in EDs for patients with symptomatic undifferentiated hypotension of nontraumatic origin. Such protocols assess left ventricular function, right ventricular size, presence of intraperitoneal fluid or pericardial effusion/tamponade, existence of abdominal aortic aneurysm, or evidence of IVC collapse [4, 5]. In this case, some of the elements of goal-directed ultrasound were used, and the care of similar patients could be improved if goal-directed ultrasound was formally adopted to guide the actions of less-experienced physicians. Several studies have shown that nonradiologist performed ultrasound has high sensitivity and specificity for hypovolemia, AAA, hemoperitoneum, and cardiac tamponade [6–9]. As these studies imply, bedside sonography is a safe, rapid, and not-difficult-to-perform way to obtain essential information for taking care of critical patients. It is especially helpful in assessing for the cause of shock and may shorten the time to treatment, though we need more studies to validate ultrasound use at ED.
